# Coping with worry while waiting for diagnostic results: a qualitative study of the experiences of pregnant couples following a high-risk prenatal screening result

**DOI:** 10.1186/s12884-016-1114-6

**Published:** 2016-10-21

**Authors:** Stina Lou, Camilla P. Nielsen, Lone Hvidman, Olav B. Petersen, Mette B. Risør

**Affiliations:** 1 DEFACTUM -Public Health & Health Services Research, Olof Palmes Alle 15, 8200 Aarhus N, Denmark; 2 Institute of Public Health, Aarhus University, Aarhus, Denmark; 3 Department of Obstetrics and Gynecology, Aarhus University Hospital Skejby, Aarhus, Denmark; 4 General Practice Research Unit, Institute of Community Medicine, UiT The Arctic University of Norway, Tromsø, Norway

**Keywords:** Pregnancy, Prenatal care, First trimester screening, Worry, Coping, Denmark, Qualitative

## Abstract

**Background:**

It is well documented that pregnant women experience increased worry and uncertainty following a high-risk prenatal screening result. While waiting for diagnostic results this worry continues to linger. It has been suggested that high-risk women put the pregnancy mentally ‘on hold’ during this period, however, not enough is known about how high-risk women and their partners cope while waiting for diagnostic results. The aim of this study was to identify the strategies employed to cope with worry and uncertainty.

**Methods:**

Qualitative, semi-structured interviews with 16 high-risk couples who underwent diagnostic testing. The couples were recruited at a university hospital fetal medicine unit in Denmark. Data were analysed using thematic analysis.

**Results:**

All couples reported feeling worried and sad upon receiving a high-risk screening result. While waiting for diagnostic results, the couples focused on coming to their own understanding of the situation and employed both social withdrawal and social engagement as strategies to prevent worry from escalating. Additionally, couples used gratitude, reassuring reasoning and selective memory as means to maintain hopes for a good outcome. Discussions about what to do in case of an abnormal test result were notably absent in the accounts of waiting. This bracketing of the potential abnormal result allowed the couples to hold on to a ‘normal’ pregnancy and to employ an ‘innocent-till-proven-guilty’ approach to their worries about the fetus’s health. None of the interviewed couples regretted having prenatal screening and all of them expected to have prenatal screening in a future pregnancy.

**Conclusions:**

The couples in this study did not put the pregnancy mentally ‘on hold’. Worry and uncertainty must be understood as managed through a diverse range of practical and emotional strategies that change and overlap in the process of waiting. Clinicians may support appropriate ways of coping with worry and waiting through empathetic and empowering clinical communication. In addition to providing adequate information and presenting options available, clinicians may support high-risk women/couples by encouraging them to seek their own personal understandings and management strategies as a way to gain some control in an uncertain situation.

## Background

The ever-advancing technologies in prenatal screening continue to provide more detailed and complex information about the fetus. This allows for early interventions and individualised care, but it also has the potential to generate acute worry in pregnant women and couples concerned about the health of their baby. Often, parents must wait for further tests and examinations, which increase the potential for worry and confusion. How best to support these women/couples continues to be a clinical challenge [1].

A high-risk screening result for chromosomal abnormalities is one example of prenatal information that requires testing and waiting for clarification. Quantitative studies have found a significant increase in anxiety following a positive screening result [2], and qualitative studies have investigated the complex information and burdensome decision-making that high-risk women face [3–5]. Invasive diagnostics (chorionic villus sampling (CVS) or amniocentesis) will provide a definite answer, but carry a small procedure-related risk of miscarriage.

Women choose diagnostic testing because they want to know the health status of their fetus [6] and because they want to stop worrying [5]. However, coming to a decision regarding invasive diagnostics does not eliminate uncertainty. What follows is a period in which the fear of miscarriage, worry about the health of the fetus and concerns about what to do in case of an abnormal result are waiting to be resolved [5]. Studies have suggested that women mentally put the pregnancy on ‘hold while’ waiting for diagnostic results [7, 8]. However, not enough is known about pregnant women and their partners’ experiences of this waiting time and the coping strategies they use to deal with uncertainty and worry.

Coping theory concerns the thoughts or actions engaged to manage stressful situations, such as avoidance, planning, seeking support or turning to religion [9, 10]. Coping theory essentially discerns between problem- and emotion-focused coping. The former is aimed at actively resolving the source of the stress, while the latter is aimed at managing the emotional distress. Though there are some studies of women’s coping following a diagnosis of fetal anomaly [11], less is known about pregnant women’s coping during the preceding diagnostic process. An understanding of this period is particularly important, because the majority of screen-positive women receive a normal diagnostic result. Thus, the unnecessary worry is the major psychosocial cost of screening for most women. Improvements in the professional support offered during this process may contribute to an appropriate management of uncertainty and worry.

Our objective was to investigate how high-risk women and their partners experience waiting for diagnostic results and to identify strategies employed to cope with worry and uncertainty.

## Methods

Qualitative methods were used to answer the research question. During a long-term anthropological study of prenatal screening at a university hospital in Denmark, high-risk pregnant women and partners were continuously recruited and interviewed by SL, who is an anthropologist. The recruitment continued until data adequacy was obtained.

### Setting

In Denmark, a combined first-trimester risk screening (cFTS) for chromosomal abnormality is available to all pregnant women as part of the standard, tax-financed prenatal care programme. In 2012, 93 % of all pregnant women in Denmark underwent cFTS [12], and generally, Danish women are knowledgeable and favourably disposed towards the cFTS [13].

At the fetal medicine unit where this study was conducted, more than 4600 cFTSs are performed every year [12]. The cFTSs are performed by sonographers (nurses and midwives certified by the Fetal Medicine Foundation, London) who do the ultrasound examination, calculate the cFTS risk estimate and inform women/couples about the result. Women at high risk (≥1:300) are counselled by the sonographers, who are trained to do this through peer-supervision programmes. All women at high risk are given the option to have an additional consultation with a fetal medicine expert or a genetic counsellor. The majority of screen-positive Danish women (85 %) choose to undergo invasive testing [14], which is normally booked the day after the cFTS. The results for trisomy 13, 18 and 21 are generally available within a week or less. Within the Danish prenatal screening programme, approximately 90 % of high-risk women who choose invasive testing receive a normal diagnostic result [14].

### Participants

**Fig. 1:**
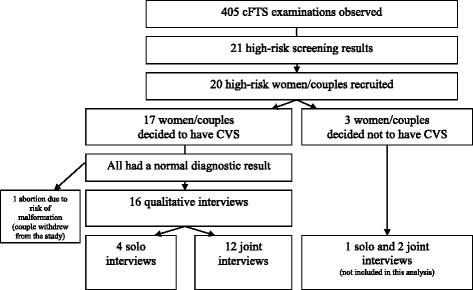
Recruitment flow chart

As a part of the overall anthropological study, SL observed more than 400 cFTSs in which 21 women/couples received a high-risk result (see Fig. 1). Seventeen couples decided to undergo diagnostic testing and consented to SL observing the procedures. All had normal diagnostic results. One couple withdrew from the study after examinations identified serious malformations in the fetus. Consequently, sixteen women/couples were interviewed by SL 2–6 weeks after the diagnostic result. Participant characteristics are shown in Table 1.

**Table 1:** Characteristics of the sample

Average age, pregnant woman (*n* = 16)	35 years (range 21–42)
Average age, partner (*n* = 16)	36 years (range 25–44)
Parity	0 = 9 (56 %)
	1 = 7 (44 %)
Married or cohabiting	16
Gestational age at interview (weeks)	Mean 18 (range 14–26)
Risk assessment	Mean 1:122 (range 1:30–1:297)
CVS	16 (100 %)
Maternal educational level^a^	
Low	2 (12 %)
Medium	7 (44 %)
High	7 (44 %)
Employed	11 (69 %)
Students	4 (25 %)
Unemployed	1 (6 %)

^a^Using the education nomenclature (ISCED) from Statistics Denmark, educational level was grouped into three categories; low (1–10 years), medium (11–14 years of education), and high (>15 years). Students are categorised by their next educational level

### Ethics and consent

Informed, oral consent was obtained prior to participant observation and informed, written consent was obtained prior to interviews. The study in general was conducted in accordance with the American Anthropology Association’s code of ethics [15]. The study was approved by the Danish Data Protection Agency (J. No. 2007-58-0010). According to Danish law, approval by the National Committee on Health Research Ethics was not required as no biomedical intervention was performed.

### Data collection

Between July 2011 and January 2012, SL conducted open-ended interviews in participants’ homes or at the hospital. Due to unforeseen circumstances, four women were interviewed without their partner. Interviews lasted 45–90 min and a semi-structured interview guide was used [16]. The interview explored the woman/couple’s expectations, experiences and considerations regarding the cFTS, the high-risk result and the CVS. Interview themes also covered woman/couple’s feelings and experiences while waiting for diagnostic results and concluded with summary questions, asking the woman/couple to reflect on the experience as a whole and on the potential impact of the high-risk experience on the pregnancy and future parenthood. Through participant observation, SL became familiar with the couples and their high-risk trajectory, which allowed specific situations and events to be explored in interviews. This triangulation of methods – participant observation and interviews – allowed opportunity to test and challenge preliminary analytical understandings.

### Data analysis

The interviews were transcribed verbatim by SL and a research assistant, and the data were analysed by SL. Upon a thorough reading of all transcripts, initial codes were generated in line with thematic analysis [17]. Both inductive (bottom-up) and deductive (top-down) codes were identified and discussed between the authors. All interviews were coded using Nvivo 9.0 software (QSR International, Doncaster, Australia). By investigating repeated patterns across the dataset and relationships between the codes, candidate themes were generated and explored in relation to the full data set. The data were scrutinized for ‘negative cases’ and contradictory evidence in order to further test the candidate themes and the preliminary analytical understandings. Throughout the analytical process, MBR acted as main supervisor and discussant, but all authors participated in the process and thus provided a forum for researcher triangulation [18], where our different professional experiences and scientific backgrounds (anthropology, medicine and political science) were used to debate and challenge the material, the analysis and the conclusions. This iterative process of defining and validating the themes continued until all authors were satisfied that there was substantial theoretical basis for explaining how women/couples at high risk cope with worry while they are waiting. To maintain the anonymity of participants, all names used in the result section are pseudonyms.

## Results

In general, the couples were very satisfied with the information they received at the cFTS. Sonographers were described as professional, empathetic and attentive in conveying the information about the high-risk result and the options available. All couples said that the decision to have CVS was their own and that they were content with their decision.

When asked about their feelings as they left the ultrasound clinic, many reported feeling empty, disappointed and sad:

‘*I felt that…. That carefree happiness was wiped out by… I wouldn’t call it grief, but it was definitely some sort of worry. Yeah… worry….*’ (Cristina, 42 years old, one child)

The high-risk result interrupted the couples’ hopes of a normal, happy pregnancy and positioned them in an intermediate state of uncertainty. When asked about their management of this period, many couples’ initially responded that there was really nothing they could do but wait. However, the subsequent dialogue revealed a range of different strategies they employed to deal with waiting and worrying.

### Managing worry by focus and distraction

An initial strategy of many of the interviewed couples was withdrawal from social relations and everyday activities. Being ‘*just us*’ was described as a safe place in difficult times. All couples stressed the importance and value of taking time to jointly attend to the uncertain situation by seeking advice, gathering information and talking it through and through.

‘*That evening, we went online to find out what a “bad” nuchal translucency looks like. You know…. Just to check that ours was OK. Sort of to confirm our own understandings that the baby was normal and digest what we had been told at the hospital.*’ (Simon, 36 years old, no children)

Though the situation was widely felt to be out of their hands, the interviewed couples took control of the situation through a process of coming to their own understanding and responses.

While withdrawing socially, the couples used TV, work and magazines as quiet entertainment to mentally disengage and to ‘*take a break*’ from worry. However, the couples simultaneously placed importance on social engagement; participating in everyday social activities, such as having a birthday party, going to lectures, or attending a music festival:

‘*We considered not going (to a niece’s birthday), but in the end it was a nice distraction. Sitting at home wouldn’t have done us any good.*’ (Oliver, 29 years old, no children)

Maintaining everyday plans and routines was experienced as valuable in shifting focus away from worry. Several couples described periods of feeling unfit or disinterested in ‘*facing the world*’, but nevertheless, they prioritised participating in ‘*normal life*’ as a strategy to prevent worry from escalating. Some couples chose not to disclose their uncertain situation as they carried on with everyday activities because this allowed them to feel normal and not be the centre of concerned attention. Others sought emotional support and advice from family and friends. In these couples’ accounts, sharing experiences and concerns helped them to re-think the situation and *keep things in perspective*. Thus, sharing was a valuable strategy for relieving worry and doubt, though sometimes it was also a source of frustration:

‘*Oh, people are so full of encouraging comments and home-spun advice, and honestly, that is the last thing you want to hear. The last thing.*’ (Anna, 34 years old, no children)

When they sought emotional support, the couples were clearly vulnerable to responses they perceived to be too empathetic, too light-hearted or otherwise misunderstood. Several couples reported becoming more strategically selective in whom to turn to for support and advice.

In summary, these women/couples coped with worry in very concrete ways using contrasting practical strategies: social withdrawal versus social engagement combined with strategies of attending to and seeking distraction from uncertainty and worry. All couples employed some combination of these strategies, alternately and sometimes even simultaneously, as when a dinner party provided a convenient distraction for a couple as well as an opportunity to talk about their worries and concerns with selected friends.

### Managing worry through reassuring reasoning

Finding ways to remain hopeful and not let worry get out of control was a main concern for the couples throughout their wait for diagnostic results. One consistent strategy was a selective recollection of the clinical communication and interactions following the high-risk screening result:

‘*I just kept thinking to myself: She [the sonographer] told us the baby looked fine. And I know it’s not rational and you can’t see the chromosomes on an ultrasound, but it was just so comforting to me and something I clung onto in those horrible days.*’ (Caroline, 30 years old, no children)

The couples actively chose to focus on clinical comments and encouragements that could be re-interpreted as hopeful and positive reassurance. Although factual information was also appreciated, reassuring comments from health professionals were considered valuable emotional leverage in coping with worry and uncertainty.

Another strategy was to reinterpret the uncertain situation in terms of the couples’ personal understandings of their pregnancy, good health and trust in a good outcome.

‘*I just got pregnant so easily. Like it was destiny or something? We kept telling ourselves that this [child] was meant to be.*’ (Eve, 38 years old, no children [solo interview])

In these personal narratives, the couples coped with worry by pragmatically emphasising positive pregnancy experiences, such as an uncomplicated pregnancy or simply ‘feeling good’. Common to these personal narratives was the tendency to support the likelihood of a good outcome. Being nauseous or having stomach pains increased worry, but this did not exclude a simultaneous use of positive personal narrative strategies. Sometimes couples referred to these interpretations as ‘irrational’ and contrary to ‘rational’ or biomedical information, and they were quick to add, ‘*this is going to sound a little crazy….*’ In these conversations, some participants mentioned having positive inner conversations with the fetus or looking for good signs in rays of sunlight. These examples illustrate how the couples engaged in practises and understandings that went beyond rational logic, but nevertheless, they regarded them as reassuring and comforting in dealing with worry and waiting.

Many couples also described how they turned to home and everyday life as a meaningful counterbalance to their high-risk status, expressing a renewed and humble awareness of all the good things that they already had in their lives: amazing children, fulfilling lifestyles and loving relationships:

‘*I tried to focus on my daughter and how blessed we are to have her.*’ (Cristina, 42 years old, one child)

Turning focus away from statistics and uncertainty towards the blessings of everyday life foregrounded everything that was *not* at risk and would persist beyond an abnormal test result. Choosing this perspective allowed the couples to create situated meaning and certainty in the midst of uncertainty and thus to create a positive counterbalance to worry.

Discussions about what to do in case of an abnormal test result were notably absent in the majority of the couples’ accounts of waiting. When asked about this during interviews, they provided two main reasons: First, the majority of couples were certain that they would terminate the pregnancy in case of an abnormal result. Second, those who expressed uncertainty about termination preferred to postpone the final decision until the final result was available. This bracketing of the potential abnormal result allowed the couples to continue and to hold on to a ‘normal’ pregnancy. Many couples described how they consciously employed an ‘innocent-till-proven-guilty’ approach to deal with their worries about the fetus’s health.

In summary, the couples coped with worry by using reassuring interpretations of the uncertain situation in accordance with their everyday lives and experiences. The strategies they employed included a selective memory of clinical comments, belief in good health combined with specific personal circumstances and a humble awareness of everyday life. Consequently, biomedical information, bodily sensations and pragmatic everyday reasoning were interlaced in the couples’ attempts to control worry and to keep up hope. Thus, drawing on the different strategies described here, each couple pieced together their own, personal puzzle of strategies to manage worry while waiting for results.

When asked to reflect on the experience of a high-risk result, the invasive testing and receiving results as a whole, the interviewed couples generally framed the situation in positive terms, highlighting the empathetic, professional approach of professionals, the speedy procedure and response, as well as the security of now knowing that the chromosomes were normal. None of the interviewed couples regretted having the cFTS and all of them expected to have cFTS in a future pregnancy.

## Discussion

Overall, our study showed that the interviewed couples employed a range of practical and emotional management strategies in order to manage uncertainty and worry while waiting for diagnostic results.

Obviously, when faced with the risk of fetal abnormality, pregnant couples become worried and perceive a loss of control. However, our results showed that the interviewed couples did not passively accept this worry or sit on their hands while waiting for clarification.

First, by withdrawing from activities to attend to the situation, gathering information and seeking support, the couples sought to manage worry by actively defining their own understandings and management strategies. Thus, even in a situation in which couples could not change or alter the final outcome, they still engaged in problem-solving coping strategies (aimed at removing or altering the stressor) [9, 10]. This process of active coping allowed them to regain some control and sense of agency in a situation in which the future was unpredictable. Our results resonate with other studies showing that pregnant women also use problem-solving coping strategies following the prenatal diagnosis of fetal anomaly, and the authors suggest that parents need opportunities for active coping following such diagnoses [11, 19]. We suggest that this is also the case for parents *waiting* for diagnosis.

Second, by positive re-interpretations of clinical information and a thankful focus on everyday life, the couples sought to infuse the uncertain situation with positive, reassuring interpretations. These responses are all types of emotion-focused coping (aimed at managing emotional distress), and Carver et al. [10] suggest that construing a stressful situation in positive terms encourages continuous, active coping. Folkman and Moskovitch [20] suggest that people under stress turn to positive, social events not only as an escape or distraction, but also as an active strategy to counterbalance the negative, emotional consequences of a stressful event.

Being at high risk is an unwelcome disruption in a pregnancy that leads to worry and concern, but as sociologist Becker reminds us, “*Disruption to life is a constant human experience.*” [21], p.180. Thus, we argue that coping with worry should be understood within a framework in which uncertainty is a generic and definitive feature of the human condition in general [21, 22]. For example, Brisch et al. [23] argue that strategies for managing worry are something that people bring with them rather than something they establish anew with every new stressful situation. Our results showed that couples were initially shocked and sad, but they were not unprepared to deal with worry and uncertainty in general. These everyday resources and strategies were crucial to the couples’ coping with waiting.

Interestingly, our findings diverge from previous research indicating that a high-risk screening result made women put the pregnancy on stand-by. Öhman et al. found that a high-risk screening result made pregnant women ‘withhold the pregnancy’ as they tried to live as if they were not pregnant [7]. Similarly, Aune and Möller suggest that high-risk women created a distance to the pregnancy as a defence mechanism to be able to handle a high-risk screening result [8]. However, understanding the wait for diagnostic results as a period when women can and will selectively ignore or ‘*withhold*’ the pregnancy does not resonate with our findings. The difference in findings may be explained by the relatively small sample sizes (of the current and the previous studies) and the different contexts (Sweden and Denmark). Furthermore, stress reactions have been shown to increase during waiting time [24] and consequently the short turnaround time in the current study might bolster positive coping and explain the differences in results. We concede that some women may employ this strategy (some of the time), but suggest a more complex approach in which worry is understood as being managed through a diverse range of practical and emotional coping strategies that change and overlap in the process of waiting for diagnostic results.

### Methodological strengths and limitations

A key strength of the present work is the inclusion of partners in the interviews and the analytical focus on joint strategies rather than gender differences (e.g., men generally being more number oriented and more optimistic [25]). This approach is consistent with other studies showing that couples experience pregnancy as a collaborative project [26, 27]. A second strength is the anthropological approach, which allowed SL to observe the couples at both the cFTS and at the CVS and added to the richness of the individual interviews and the analyses. Furthermore, this sampling strategy resulted in a high response rate and low selection bias.

To evaluate the results of this study, some considerations must be taken into account. First, the couples were given the result of the cFTS immediately after the ultrasound scan. Several studies have documented the positive effect of ultrasound on maternal anxiety [28] and fetal–maternal attachment [29]. In the present study, all couples had normal ultrasound examinations, which may have increased their ability to control worry and nurture positive thinking. Couples with a visibly affected fetus may have more difficulties with controlling worry. Second, by the time they were interviewed, all of the participants had received a normal diagnostic result, which may have influenced their memory. Because interviews were conducted 3–13 weeks after the CVS procedure, some recall bias is possible. However, in interviews the couples seemed to remember the situation vividly, which is consistent with research showing that the recall of emotional, pregnancy-related events is highly consistent over time [30]. Third, the average age of women in this study (35 years) is higher than the national average age for women giving birth (30.9 years) [31]. Younger women and couples might have different perspectives and experiences.

Finally, Denmark was the first country to offer free, tax-financed prenatal screening to all pregnant women. Thus, pregnant women are very familiar with the availability of the cFTS, and the knowledge about the procedure is relatively high, which correlates with lower decisional conflict [32]. Moreover, the study was conducted in the fetal medicine unit of a university hospital that routinely conveys such information and has the appropriate expertise to communicate sensitively with high-risk couples. Consequently, the couples in this sample may have felt more involved, informed and empowered, and thus less worried than high-risk couples in other settings.

## Conclusions

The present study shows that high-risk screening results generated both worry and uncertainty. However, the couples actively pieced together personal coping strategies that helped them stay positive and counterbalance worry. None of the interviewed couples reported putting the pregnancy ‘on stand-by’. None of the couples regretted having the cFTS and all of them expected to have cFTS in a future pregnancy.

In addition to providing adequate information and presenting options available, clinicians can effectively support high-risk women/couples. By addressing different coping strategies, clinicians can encourage couples to seek their own personal understandings and management strategies as a way to gain some control in an uncertain situation. Clinicians may suggest to the couples the importance of maintaining everyday activities while also taking time out for contemplation and withdrawal. Existential uncertainties and worries are intrinsic to a high-risk status and by addressing them as normal and manageable, clinicians may encourage women not to put the pregnancy on hold. Furthermore, by adopting a reassuring attitude and sharing positive information (e.g. by underscoring chances of a normal outcome or by other small gestures of encouragement), clinicians may support an appropriate way of coping with worry and waiting.

## Abbreviation

cFTS: Combined first trimester screening
